# Pharmacological and non-pharmacological prevention and management of delirium in critically ill and palliative patients in the inpatient setting: a review

**DOI:** 10.3389/fmed.2024.1403842

**Published:** 2024-07-17

**Authors:** Leah Chan, German Corso

**Affiliations:** Saint James School of Medicine, Park Ridge, IL, United States

**Keywords:** delirium, pharmacological interventions, management – intensive care, delirium prevention and management, agitation, non-pharmacologic interventions

## Abstract

**Introduction:**

This review explores delirium in critically ill patients in the inpatient setting, focusing on its prevention and management. It evaluates the efficacy of both current pharmacological and non-pharmacological interventions, aiming to provide a comprehensive overview.

**Methods:**

A systematic literature search was conducted to identify relevant studies investigating the prevention and management of delirium resulting in a final sample of 26 articles for analysis.

**Results:**

Of the 26 articles analyzed for this review (*N* = 8,831 participants) of controlled trials, 16 studies examined the prevention of delirium, 9 explored the treatment of delirium, and 1 investigated both prevention and treatment of delirium.

**Discussion:**

Among the reviewed studies, there is evidence that non-pharmacologic methods are effective in the prevention of delirium. Evidence regarding pharmacological interventions for delirium prevention is varied and inconclusive, with some indication that atypical antipsychotics like aripiprazole and quetiapine may reduce the incidence of delirium. Regarding the treatment of delirium, there is limited evidence supporting the use of pharmacological agents. Additional double-blinded, randomized, placebo-controlled clinical trials are needed to investigate the efficacy of pharmacologic agents for diverse hospitalized populations.

## Introduction

Delirium is a debilitating neuropsychiatric syndrome often encountered in patients requiring inpatient care, causing significant distress (1). According to the Diagnostic and Statistical Manual of Mental Disorders, DSM-5, delirium is defined as, “disturbance in attention accompanied by reduced awareness of the environment that develops over a short period, representing a change from baseline attention and awareness that tends to fluctuate in severity throughout the day and is associated with additional disturbances in cognition that are not better explained by another pre-existing, established or evolving neurocognitive disorder, and do not occur in the context of a severely reduced level of arousal, and evidence from the history, physical examination or laboratory findings that indicate that the disturbance is a direct physiological consequence of another medical condition, substance intoxication, or withdrawal” (2). It manifests with disruptions in cognition, attention, and consciousness, often accompanied by symptoms such as hallucinations, illusions, and agitation (1, 3). A diagnosis of subsyndromal delirium can be made when not all DSM-5 criteria are met (4, 5). Delirium may present as either hypoactive (lethargy, decreased responsiveness), hyperactive (agitation, restlessness), or mixed hypoactive/hyperactive where patients alternate between both presentations (4).

The exact pathophysiology of delirium is not well understood, but it is likely a consequence of the disruption of various pathways implicated in normal cognitive function, particularly during critical illness. Many hypotheses have been proposed, including neuroinflammation, compromised cerebral blood flow, and neurotransmitter dysregulation (3, 4).

Delirium affects a substantial proportion of hospitalized patients with prevalence rates ranging from 7 to 50% in medical and surgical units and reaching as high as 82% in intensive care units (ICUs) (1). Its clinical significance is underscored by its association with various adverse outcomes. This includes prolonged hospital stays, cognitive and functional deterioration, increased morbidity, and increased mortality rates (1, 3, 4). Delirium is independently associated with an increased risk of death (3, 4). Notably, delirium independently elevates the risk of mortality (3, 4), extending beyond the acute hospitalization period and resolution of symptoms to 1 year post-discharge from the ICU (6).

Traditionally, the treatment approach for delirium and agitation focuses on addressing underlying medical causes supplemented by a range of pharmacological (e.g., antipsychotics) and non-pharmacological interventions (1, 3). Historically, haloperidol was the preferred pharmacological agent for treatment, but concerns over its safety and side effects led to the adoption of second-generation (atypical) antipsychotics, which offer comparable efficacy with improved safety profiles (3). Other medication classes, including alpha-2-agonists, acetylcholinesterase inhibitors, antidepressants, and benzodiazepines, have also been explored for delirium management (1), yet conflicting perspectives persist regarding their effectiveness (3), prompting interest in non-pharmacological strategies (3, 7).

The ABCDEF bundle is comprised of six components. The first component emphasizes Assessing, preventing, and managing pain, recognizing its potential to precipitate delirium (3, 7). This is also the basis for a study discussed later in this review, which investigated the use of pain medications such as opioids in delirium treatment (7). The second component involves both spontaneous awakening trials (SATs) and spontaneous breathing trials (SBTs), aiming to reduce mechanical ventilation duration and ultimately enhance ICU outcomes (3). This is also the basis of several studies’ secondary outcome measurements of time to mechanical ventilation cessation or number of days mechanically ventilated (8–15). The choice of analgesia and sedation constitutes the third element. The fourth component focuses on delirium: assessment, prevention, and management emphasizing early detection using validated screening methods like the Pain, Agitation/Sedation, Delirium, Immobility, and Sleep Disruption in Adult Patients in the ICU (PADIS) Guideline recommended Confusion Assessment Method for the ICU (CAM-ICU) or the Intensive Care Delirium Screening Checklist (ICDSC) (4), and a thorough search of potential aetiologies, such as infection (3). The fifth component involves early mobility and exercise, known to enhance the likelihood of returning to baseline function post-discharge. The final part of the bundle emphasizes family engagement and empowerment, which positively impact ICU-related outcomes (3), as explored in three studies examining the role of family support in delirium (16–18). Delivery of the complete ABCDEF bundle has shown significant benefits for delirium-related outcomes (3, 19).

Given the inconsistent outcomes of pharmacological treatments for delirium, there is a shift in emphasis towards prevention and early detection. Prevention strategies encompass both non-pharmacological and pharmacological interventions, with the Society of Critical Care Medicine (SCCM) guidelines advocating strongly for non-pharmacological approaches. These include mitigating risk factors (e.g., promoting healthy sleep patterns), tailoring analgesia and sedation individually (e.g., avoiding benzodiazepines), and promoting patient mobility (3). The ABCDEF bundle has demonstrated significant benefits not only in delirium treatment but also in its prevention (3, 19). Additionally, considering the neurotransmitter-related hypotheses of delirium, pharmacological prevention strategies utilizing drugs commonly employed in delirium treatment have undergone further investigation. However, like pharmacological management, the efficacy of pharmacological prevention methods for delirium shows varied outcomes, necessitating continued research (4).

Numerous reviews and trials addressing the efficacy of delirium treatment and prevention strategies often exhibit limitations, typically focusing on specific drug classes, patient cohorts, or clinical settings, thereby lacking generalizability. In this review, we explore the effects of a broad array of pharmacological and non-pharmacological interventions on delirium prevention and management within critically ill and palliative care populations, aiming to provide insights applicable across diverse clinical contexts.

## Methods

A systematic literature review was performed to evaluate the efficacy of pharmacological and non-pharmacological interventions on delirium prevention and management following the Preferred Reporting Items for Systematic Reviews and Meta-Analyses (PRISMA) guidelines (20).

In February 2024, systematic searches of electronic databases, such as PubMed, were performed by combining keywords related to delirium prevention and management in the critically ill and palliative population, such as “delirium,” “confusion,” “agitation,” “palliative,” “hospice,” “end of life,” “critical,” “critically ill,” “terminal,” “advanced,” “prevention,” “treatment,” and “management” with Boolean operators, such as “AND” and “OR,” to identify relevant studies reporting the effectiveness of pharmacological and non-pharmacological strategies on the prevention and treatment of delirium.

The following inclusion criteria were used: (1) a scholarly reviewed journal/source such as PubMed, (2) articles published within the past 10 years, (3) published in the English language, (4) randomized or non-randomized clinical trials (RCT or non-RCT), (5) studies that included human participants, and (6) assessed a pharmacological, or non-pharmacological intervention to delirium related symptoms. The exclusion criteria consisted of (1) observational studies, (2) reports without outcome data, (3) feasibility studies and methods papers, (4) single-arm intervention studies, and (5) studies conducted in the outpatient setting.

Identified articles underwent filtering using automated software and were subsequently imported into a spreadsheet program. After eliminating duplicates, each article underwent initial screening based on title and abstracts, followed by a full-text review to determine inclusion and exclusion criteria. Data from the studies, including participant numbers, settings, intervention types, and other relevant details, were extracted and organized in the spreadsheet program. Information from all included articles was recorded and compiled for comprehensive review (Table 1).

**Table 1:** Characteristics of intervention trials included in this review.

Authors	Year	Journal	Research focus (prevention/treatment)	Type of study	Sample size	Setting	Intervention type	Medications and controls	Assessment tools for delirium and delirium related outcomes	Conclusion
van den Boogaard et al. (21)	2018	JAMA	Prevention	Randomized controlled trial	1789	ICU	Pharmacological	Haloperidol	CAM-ICU, ICDSC, RASS	The incidence of delirium was not reduced, nor was survival among the critically ill improved in patients treated with haloperidol.
Al-Qadheeb et al. (8)	2016	Critical Care Medicine	Prevention	Randomized controlled trial	68	Medical and surgical ICU	Pharmacological	Haloperidol	ICDSC	Early initiation of low-dose intravenous haloperidol did not prevent the conversion of subsyndromal delirium to delirium in mechanically ventilated, critically ill ICU patients.
Mokhtari et al. (22)	2020	European Journal of Clinical Pharmacology	Prevention	Randomized controlled trial	53	Neurosurgical ICU	Pharmacological	Aripiprazole	CAM-ICU	Aripiprazole is effective in the prevention of delirium in the neurosurgical ICU.
Abraham et al. (9)	2021	The Surgeon	Prevention	Randomized controlled trial	71	Surgical trauma ICU	Pharmacological	Quetiapine	CAM-ICU	Low-dose quetiapine given on a schedule is effective as prophylaxis for delirium in high-risk, surgical trauma ICU patients.
Hollinger et al. (23)	2021	Journal of Clinical Anesthesia	Prevention	Randomized controlled trial	182	Inpatient preoperative care	Pharmacological	Ketamine, Haloperidol	MMSE, DOS, NuDESC, ICDSC	The study findings do not support the use of ketamine, haloperidol, or a combination or both for preventing postoperative delirium.
Su et al. (24)	2016	The Lancet	Prevention	Randomized controlled trial	700	Surgical ICU	Pharmacological	Dexmedetomidine	CAM-ICU, RASS	During the first 7 days after non-cardiac surgery, low dose dexmedetomidine is effective at decreasing the incidence of delirium.
Lee et al. (10)	2020	Transplantation Proceedings	Prevention	Randomized controlled trial	201	Surgical ICU	Pharmacological	Dexmedetomidine	CAM-ICU	Intraoperative and early postoperative low-dose dexmedetomidine infusion did not decrease delirium incidence or duration after living-donor liver transplant.
Wibrow et al. (11)	2022	Intensive Care Medicine	Prevention	Randomized controlled trial	841	ICU	Pharmacological	Melatonin	CAM-ICU	Melatonin given within 48 h of ICU admission is not effective at preventing delirium.
Potharajaroen et al. (25)	2018	Psychiatry Research	Prevention	Randomized controlled trial	62	Surgical ICU	Non-pharmacological		CAM-ICU	The incidence of delirium in critically ill surgical patients admitted to the SICU was significantly reduced in bright light and oxygen therapy treated patients. Compared to standard care, bright light therapy significantly protected against delirium onset.
Faustino et al. (12)	2022	Journal of Critical Care	Prevention	Randomized controlled trial	144	ICU	Non-pharmacological		CAM-ICU, RASS	Compared to standard care, combined non- pharmacological interventions were more effective in reducing the incidence of delirium in critically ill patients.
Contreras et al. (16)	2021	Revista gaúcha de enfermagem	Prevention	Randomized controlled trial	81	ICU	Non-pharmacological		CAM-ICU	Implementing comprehensive nursing interventions such as spatial and temporal guidance, visual stimulus, auditive stimulus, and family support effectively reduced the incidence of delirium in critically ill patients.
Munro et al. (17)	2017	Heart and Lung	Prevention	Randomized controlled trial	30	ICU	Non-pharmacological		CAM-ICU	Automated, scripted messages reduced delirium in critically ill adults, with family voices being more effective than unknown ones.
Kasapoğlu and Enç. (18)	2022	Geriatric Nursing	Prevention	Randomized controlled trial	94	General and pulmonary ICU	Non-pharmacological		RASS, CAM-ICU	Multicomponent-non-Pharmacological Nursing Interventions, such as daily newspaper reading, listening to orientation messages, and wearing eye patches were more effective at decreasing the incidence of delirium in critically ill patients than standard care.
Dong et al. (26)	2020	Annals of Palliative Medicine	Prevention	Randomized controlled trial	106	Inpatient	Non-pharmacological		CAM, MDAS, SPMSQ	Implementing a multidisciplinary comprehensive intervention model based on the improved Hospital Elderly Life Program effectively prevented and reduced severity of delirium in patients with severe acute pancreatitis.
Brennan et al. (27)	2023	Australian Critical Care	Prevention	Randomized controlled trial	2,566	ICU	Non-pharmacological		RASS, CAM, CAM- ICU	Introduction of non-pharmacological nurse-led interventions did not decrease the incidence and duration of delirium among adults admitted to intensive care.
Liang et al. (28)	2023	Nursing in Critical Care	Prevention	Randomized controlled trial	152	Surgical ICU	Non-pharmacological		CAM-ICU	The statistical significance of preventing delirium through sensory stimulation was not observed. However, the intervention group showed significantly reduced duration and severity of delirium, along with a notable increase in delirium-free days.
Page et al. (13)	2017	Lancet Respir Med	Both	Randomized controlled trial	142	ICU	Pharmacological	Simvastatin	CAM-ICU, RASS	Early administration of simvastatin did not show significant efficacy in preventing or treating delirium in critically ill patients undergoing mechanical ventilation.
Smit et al. (14)	2023	Critical Care	Treatment	Randomized controlled trial	132	Mixed medical/surgical ICU	Pharmacological	Haloperidol	CAM-ICU, RASS	Haloperidol did not reduce delirium and coma in critically ill patients with delirium.
Hui et al. (29)	2020	Lancet Oncology	Treatment	Randomized clinical trial	68	Palliative care unit	Pharmacological	Haloperidol, chlorpromazine, haloperidol and chlorpromazine combination	RASS, MDAS	Though not statistically significant, three strategies of neuroleptics: (1) haloperidol dose escalation, (2) neuroleptic rotation to chlorpromazine, or (3) combination therapy with haloperidol and chlorpromazine may be effective in rapidly reducing restlessness and agitation in the last days of life.
Agar et al. (30)	2017	JAMA Internal Medicine	Treatment	Randomized controlled trial	247	Inpatient hospice and hospital palliative care	Pharmacological	Haloperidol, Risperidone	NuDESC, MDAS	The placebo group was more effective at decreasing delirium symptoms and severity and required less use of rescue midazolam when compared to haloperidol and risperidone.
van der Vorst et al. (31)	2020	The Oncologist	Treatment	Randomized controlled trial	98	Medical oncology ward or high-care hospice facility	Pharmacological	Olanzapine, Haloperidol	DRS-R-98	When compared with haloperidol, treatment with olanzapine was not statistically significant different regarding delirium-related outcomes.
Hui et al. (32)	2017	JAMA	Treatment	Randomized controlled trial	90	Acute palliative care unit	Pharmacological	Lorazepam and haloperidol, haloperidol	RASS, MDAS	Adding lorazepam to haloperidol resulted in a greater reduction in agitated delirium in patients with advanced cancer when compared with haloperidol alone.
Ferraz Gonçalves et al. (33)	2016	Journal of Pain and Palliative Care Pharmacotherapy	Treatment	Place randomized trial	79	Palliative care unit	Pharmacological	Midazolam and Haloperidol, Haloperidol	Short CAM	Using haloperidol and midazolam in combination was more effective in the treatment of agitation in delirium than haloperidol alone.
Carrasco et al. (34)	2016	Critical Care Medicine	Treatment	Nonrandomized controlled trial	132	Medical/surgical ICU	Pharmacological	Dexmedetomidine	CAM-ICU, ICDSC, RASS	Dexmedetomidine demonstrated efficacy as a rescue medication for managing agitation resulting from delirium in non-intubated patients, particularly when haloperidol proves ineffective.
Khan et al. (15)	2019	Journal of the American Geriatric Society	Treatment	Randomized clinical trial	351	Medical, surgical and progressive ICU	Pharmacological	Multi-component intervention targeting three neurotransmitters (acetylcholine, dopamine, and GABA)	RASS, CAM-ICU, CAM-ICU-7, DRS- R-98	The multi-component pharmacological management of delirium bundle did not reduce delirium duration and delirium severity.
Habiger et al. (7)	2016	Behavioral Neurology	Treatment	Randomized controlled trial	352	Nursing home	Pharmacological	Opioids, Anticonvulsants	NPI-NH	Pain appeared to be an underlying cause of psychosis and agitation. Effective pain management was associated with reduced psychosis and agitation symptoms in patients with advanced dementia.

## Results

### Search

**Figure 1:**
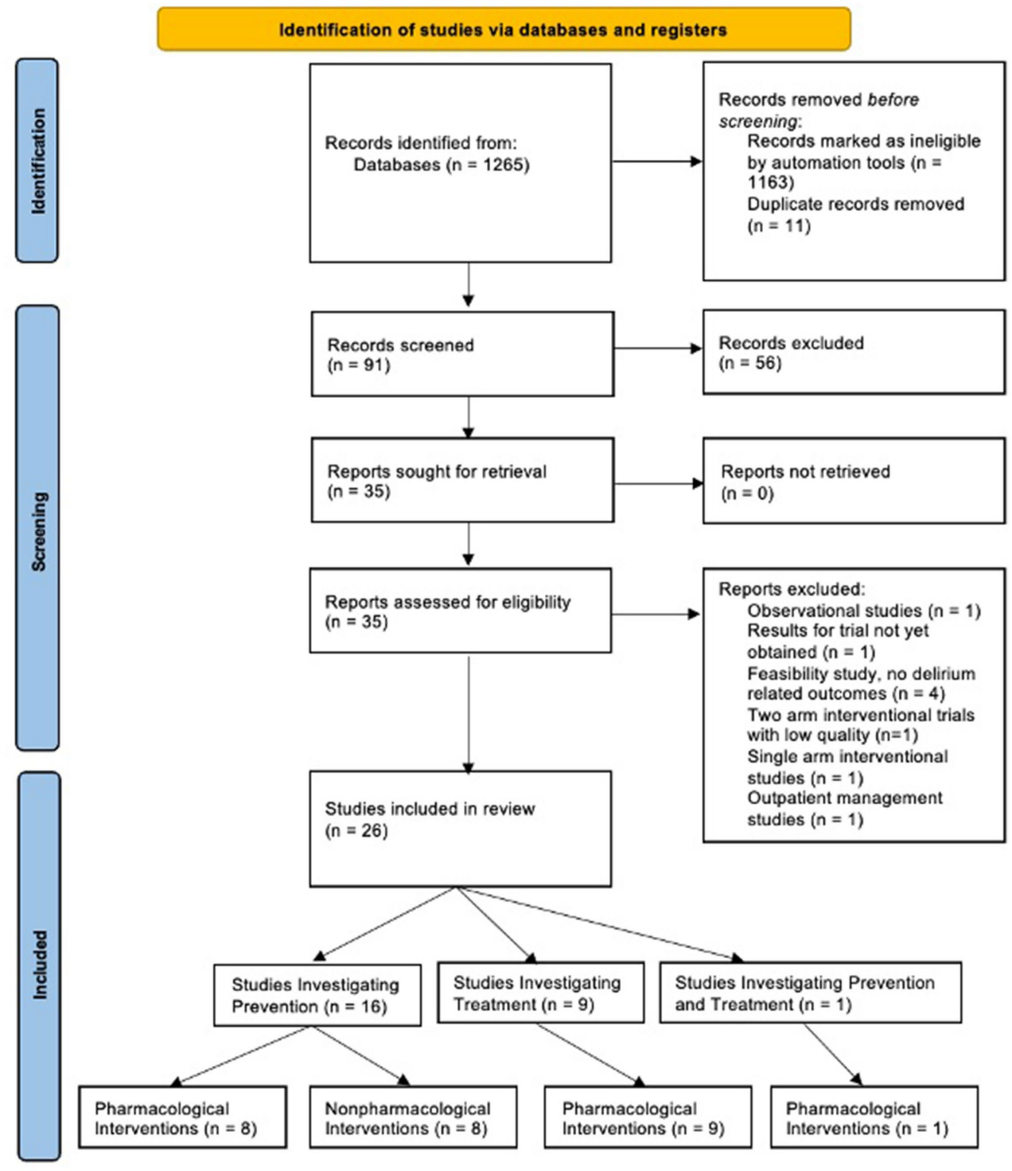
PRISMA diagram revealing search results and reasons for exclusion.

**Figure 2:**
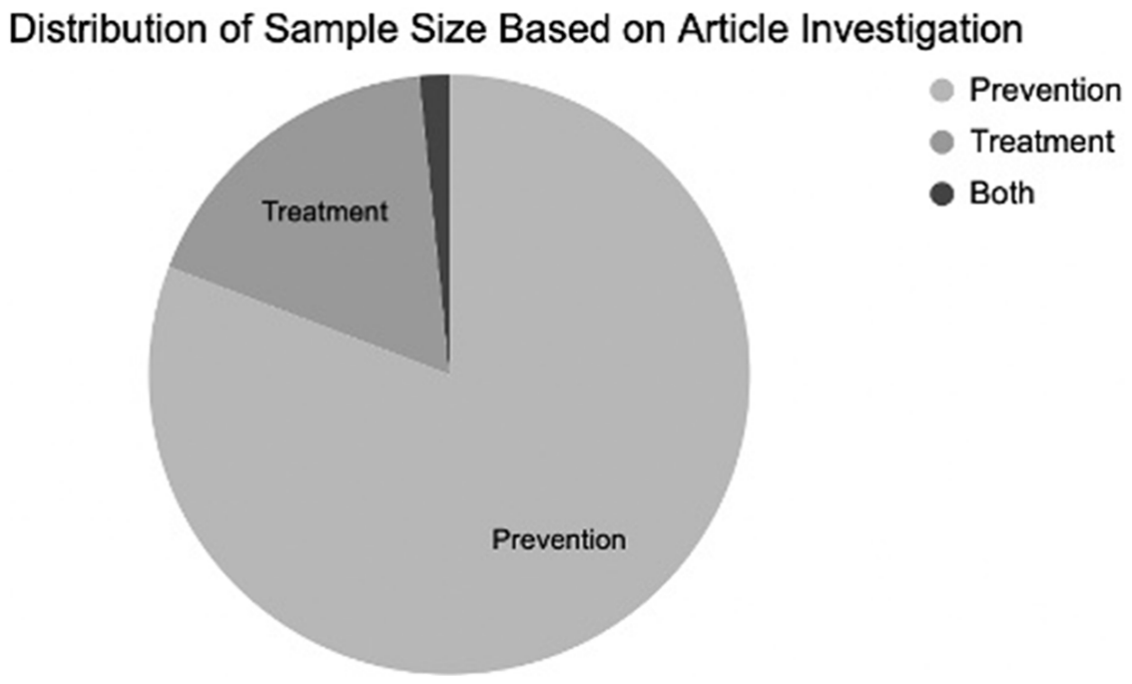
Distribution of sample size based on article investigation on treatment or prevention.

Systematic searches yielded 1,265 articles. Of these, 1,163 were excluded by automation tools that filtered for factors such as study design, publication date, and language; leaving 91 remaining articles to be screened. Of the 91 articles screened by title and abstract, 56 were excluded. Of the 35 remaining articles, 9 were removed after a full-text review, leaving a total of 26 articles (*N* = 8,831 participants) that met all criteria and were included in the review (Figure 1). Figure 2 shows the distribution of the number of patients included in this review based on investigations on prevention versus treatment.

**Figure 3:**
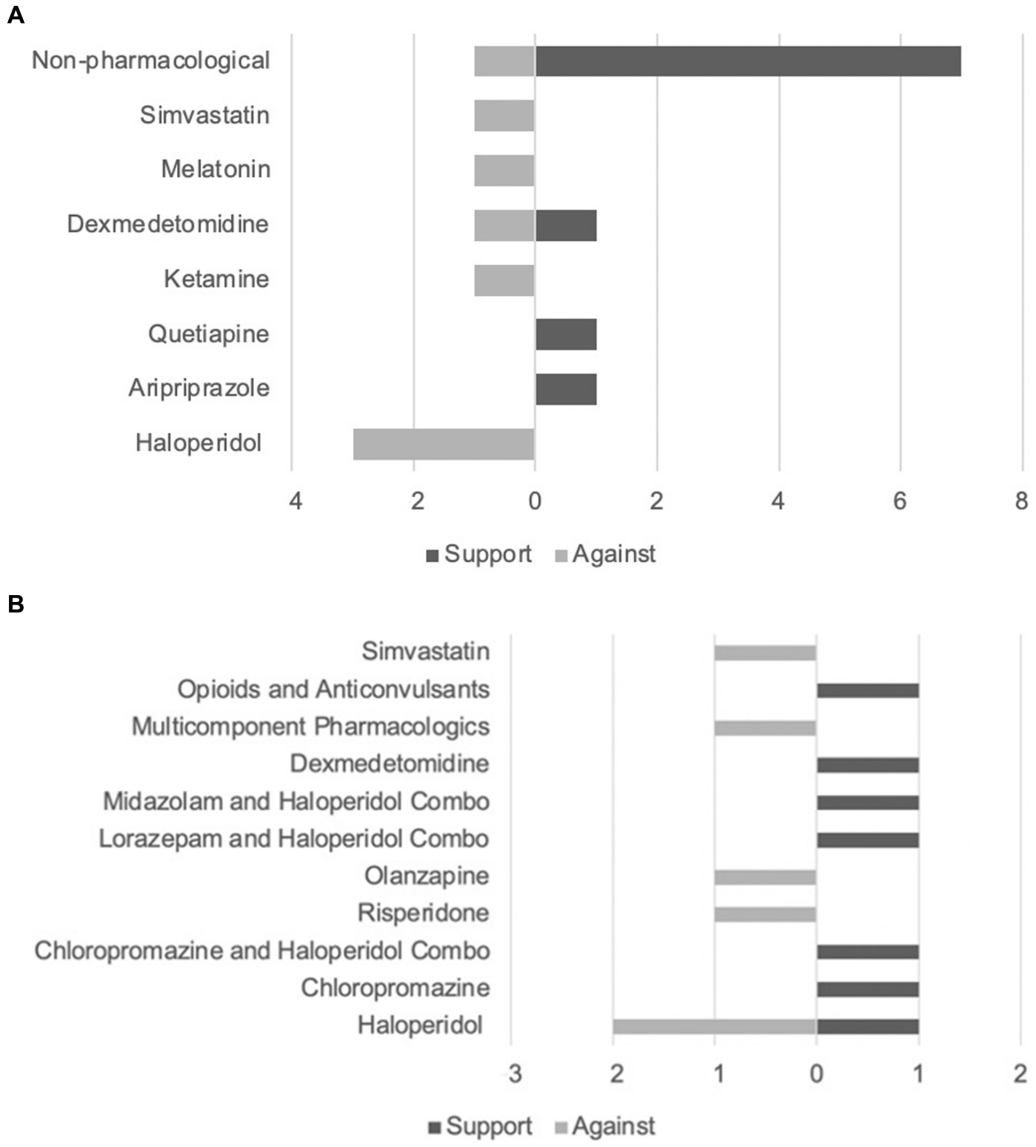
**(A)** Evidence supporting and opposing delirium prevention techniques. **(B)** Evidence supporting and opposing delirium treatment techniques.

The majority (*N* = 16) of the included articles investigated the prevention of delirium using pharmacological (8–11, 13, 21–24) (*N* = 9) and non-pharmacological (12, 16–18, 25–28) (*N* = 8) methods. Of the pharmacological prevention methods, the following medications were studied: antipsychotics (8, 9, 21–23) (*N* = 5), ketamine (23) (*N* = 1), alpha-2-agonists (10, 24) (*N* = 2), melatonin (11) (*N* = 1), and simvastatin (investigating both prevention and treatment of delirium) (13) (*N* = 1). The remaining articles investigated the different pharmacological treatments for delirium (7, 13–15, 29–34) (*N* = 10) and included: antipsychotics (14, 29–33) (*N* = 6), benzodiazepines (32, 33) (*N* = 2), alpha-2-agonists (34) (*N* = 1), opioids (7) (*N* = 1), anticonvulsants (7) (*N* = 1), analgesics (*N* = 1), and multi component interventions targeting three neurotransmitters (acetylcholine, dopamine, and GABA) (15) (*N* = 1). Figures 3A,B summarize the evidence supporting and opposing each method of intervention.

### Assessments of delirium and related symptoms

Among the included trials, the diagnosis and outcome measurement of delirium and delirium-related symptoms were observed using a broad assortment of assessment tools. The following delirium assessment tools were used: Confusion Assessment Method (CAM) and/or Confusion Assessment Method for Intensive Care Unit (CAM-ICU) and/or Short Confusion Assessment Method (Short CAM) (9–18, 21, 22, 24–28, 33, 34) (*N* = 19), CAM-ICU-7 (15) (*N* = 1), Intensive Care Delirium Screening Checklist (ICDSC) (8, 21, 23, 34) (*N* = 4), Delirium Rating Scale-Revised-98 (DRS-R-98) (31) (*N* = 1), Memorial Delirium Assessment Scale (MDAS) (26, 29, 30, 32) (*N* = 4), Nursing Delirium Screening Scale (NuDESC) (23, 30) (*N* = 2), Richmond Agitation and Sedation Scale (RASS) (12–15, 18, 21, 22, 24, 27, 29, 32, 34) (*N* = 12), Mini Mental Status Examination (MMSE) (23) (*N* = 1), Delirium Observation Scale (DOS) (23) (*N* = 1), Short Portable Mental Status Questionnaire (SPMSQ) (26) (*N* = 1), and Neuropsychiatric Inventory Nursing Home Version (NPI-NH) (7) (*N* = 1).

### Risk of bias assessment

**Figure 4:**
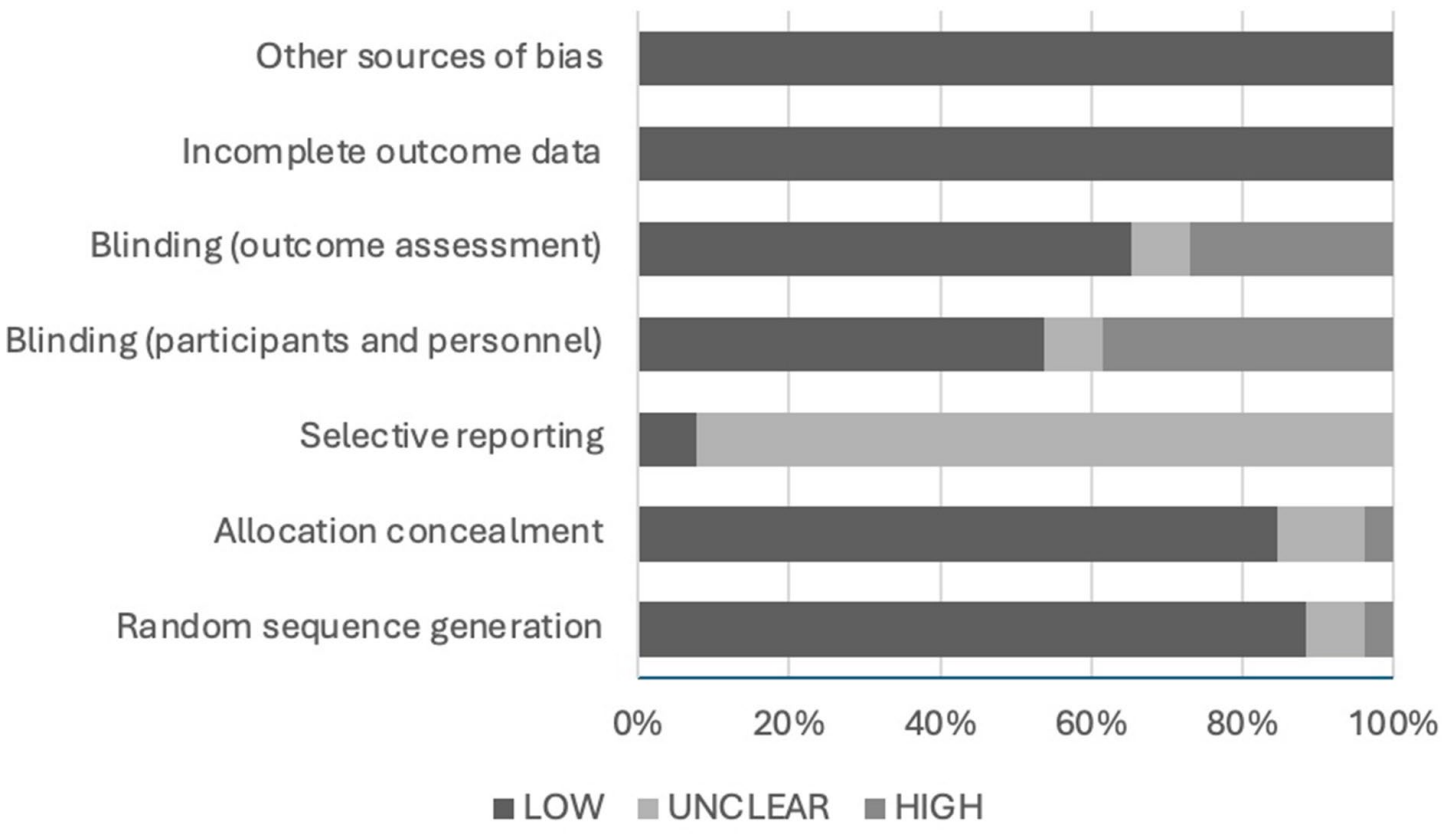
Risk of bias of included articles.

The risk of bias was assessed using the Cochrane Risk of Bias Assessment Tool. The results of the assessment are demonstrated in Figure 4. The risk of bias related to random sequence generation was low in 88.5% (*N* = 23) of the articles, unclear in 7.7% (*N* = 2), and high in 3.8% (*N* = 1) of the included articles. The risk of bias due to allocation concealment was low in 84.6% (*N* = 22), unclear in 11.5% (*N* = 3), and high in 3.8% (*N* = 1) of the included articles. The risk of bias associated with selective reporting was unclear in 92.3% (*N* = 24) and low in 7.7% (*N* = 2) of the articles. The chance of bias correlated to blinding of participants and personnel was low in 53.8% (*N* = 14) of the articles, unclear in 7.7% (*N* = 2), and high in 38.5% (*N* = 10) of the included articles. The likelihood of bias due to blinding of the outcome assessment was low in 65.4% (*N* = 17) of the articles, unclear in 7.7% (*N* = 2), and high in 26.9% (*N* = 7) of the articles. Lastly, the risk of bias due to incomplete outcome data or any other source was low in all included articles (*N* = 26).

### Pharmacological prevention

#### Antipsychotics

Antipsychotics, particularly haloperidol, are among the most extensively studied pharmacological agents for preventing and managing delirium (1). In this review, two double-blinded, placebo-controlled randomized controlled trials exclusively investigated the prophylactic effects of haloperidol (8, 21), while another placebo-controlled, double-blinded randomized controlled trial compared haloperidol with ketamine (23). Additionally, randomized studies examining aripiprazole (22) and quetiapine (9) were also included for analysis.

Between July 2013 and March 2017, a double-blinded, placebo-controlled randomized controlled trial assessed the effectiveness of prophylactic haloperidol in 1,789 participants across 21 ICUs. Participants were randomized into three groups: one receiving 1 mg of haloperidol every 8 h, another receiving 2 mg of haloperidol every 8 h, and a control group receiving a placebo (sodium chloride 0.9%) every 8 h, with comparable PRE-DELIRIC scores of 26.3, 26.1, and 25.6, respectively. However, the intervention group receiving 1 mg of haloperidol was prematurely discontinued due to inefficacy while still blinded. The incidence of delirium between the haloperidol group (33.3%) and the placebo group (33.0%) did not differ statistically. Similarly, there were no significant differences in the number of delirium-free days (28 for both groups) or median days to delirium occurrence (3 for both groups). Additionally, the percentage of patients requiring physical restraints did not differ significantly between groups: 27.0% in the haloperidol group and 24.8% in the placebo group. Furthermore, rates of extrapyramidal side effects, safety concerns (such as QTc prolongation), and serious adverse events did not significantly differ between 1 mg haloperidol, 2 mg haloperidol and control groups, with differences at most reaching 0.4, 0.6, and 0.2%, respectively. In conclusion, among critically ill adults at high risk of delirium, prophylactic haloperidol compared with placebo did not yield statistically significant differences across all measures, challenging the efficacy of haloperidol in delirium prevention (21).

Similarly, Al-Qadheeb et al. (8) investigated the efficacy of haloperidol in preventing the progression to delirium in patients with subsyndromal delirium through a double-blind, placebo-controlled pilot study. This trial, conducted across 3 ICUs, involved 68 mechanically ventilated patients randomized by a computer generator to receive either 1 mg of IV haloperidol every 6 h or a placebo (5% dextrose in water) every 6 h. Baseline characteristics, including PRE-DELIRIC score and ICDSC score, were comparable between groups, differing by only 3% and 0, respectively. Delirium was assessed using the ICDSC and psychiatric confirmation. Results showed that 35.3% of participants in the haloperidol intervention group, compared to 23.5% in the placebo group, developed delirium, with an average duration of 2 and 3 days, respectively. Haloperidol-treated subjects exhibited fewer hours per day of agitation compared to placebo-treated subjects (0 vs. 2). Additionally, the proportion of participants experiencing adverse events or requiring medication discontinuation due to safety concerns (including QTc interval prolongation: 11.8% [haloperidol] vs. 2.9%; extrapyramidal symptoms: 2.9% [haloperidol] vs. 0%; excessive sedation: 2.9% [haloperidol] vs. 0%; hypotension: 2.9% [haloperidol] vs. 2.9%) did not differ significantly between groups (*p* > 0.05). These findings suggest that early administration of haloperidol did not prevent the conversion to delirium in patients with subsyndromal delirium. However, haloperidol may play a role in reducing delirium-related symptoms such as agitation (8).

The scarcity of literature regarding atypical antipsychotics as prophylaxis for delirium prompted a 2017 double-blinded, randomized, placebo-controlled study evaluating aripiprazole’s efficacy in preventing delirium in the neurosurgical ICU. In this study (*N* = 53), participants were randomized into a treatment group receiving 15 mg enteric aripiprazole for 7 days or a placebo group receiving identical-looking tablets for 7 days. Both groups also received non-pharmacological delirium prevention based on ICU protocols. Baseline characteristics, such as RASS score, were comparable between groups, differing by a maximum of 5% in those scoring −1 and 1 at baseline. Using the CAM-ICU tool for delirium assessment, the incidence of delirium in the intervention and control groups was 20 and 55%, respectively. The comparison of days to delirium onset was not statistically significant (2.17 ± 0.41 days in the aripiprazole group compared to. 2.09 ± 0.30 days in the placebo group). Additionally, there were no statistically significant serious adverse reactions related to aripiprazole observed during the study. Although three patients (one in the aripiprazole group and two in the placebo group) experienced QTc prolongation, these results were not statistically significant (*p* = 0.548). Overall, the analysis concluded that enteric aripiprazole administration in ICU patients effectively prevented delirium, with minimal observed side effects or safety concerns (22).

Abraham et al. (9) investigated the potential pharmacological prophylaxis for delirium using the atypical antipsychotic, quetiapine. The trial included 71 high-risk patients admitted to the surgical ICU, divided into an intervention group receiving 12.5 mg of quetiapine every 12 h (*N* = 22) and a control group receiving no pharmacological intervention (*N* = 49). Baseline characteristics between groups were generally similar, except for the average PRE-DELIRIC score (71.3% for the control group and 66.4% for the intervention group), though not statistically significant (*p* = 0.44). The incidence of delirium was significantly higher in the control group (77.6%) compared to the quetiapine group (10%) (*p* = 0.008). The mean time to onset of delirium in the control group was 1.41 and 2.53 days in the quetiapine group. The average delirium duration was greater in the control group (2.3 days in the control group compared to 1.0 days in the quetiapine group), though these results were not statistically significant (*p* = 0.06 and 0.52, respectively). No significant adverse effects were reported, except for QTc prolongation >500 ms, which was more predominant in the quetiapine group (41.7%) than in the control group (9.4%). Overall, the study provided evidence that scheduled quetiapine is efficacious as prophylaxis for delirium in high-risk critically ill trauma and surgical patients (9).

#### Ketamine

As previously noted, haloperidol is extensively studied in the context of delirium revealing contradictory findings (1, 23). Similarly, uncertainty surrounds the use of ketamine for delirium. The Baden PRIDe (Prevention and Reduction of Incidence of postoperative Delirium) study, conducted from July 2013 to December 2018, aimed to assess whether postoperative delirium and cognitive impairment could be prevented using ketamine, haloperidol, or a combination of both through a randomized, placebo-controlled, double-blinded clinical trial. The trial included 182 participants divided into four groups: placebo (*N* = 44); haloperidol 5 μg/kg BW intervention (*N* = 45); ketamine 1 mg/kg BW intervention (*N* = 47); and haloperidol 5 μg/kg BW plus ketamine 1 mg/kg BW (*N* = 46). Participants received the intervention or placebo once just before anesthesia induction and were followed for 3 days post-surgery. Baseline characteristics and anesthesia methods were comparable across all four groups. Delirium and cognitive impairment were assessed using various methods (MMSE, DOS, NuDESC, and ICDSC). The incidence of postoperative delirium was 11.1% in patients receiving haloperidol, 6.4% in patients receiving ketamine, 4.3% in patients receiving haloperidol plus ketamine, and 9.1% in patients receiving placebo. None of the interventional study arms were statistically superior to the placebo group in preventing postoperative delirium (*p* = 0.39). Similarly, postoperative cognitive impairment occurred in 15.6% of patients receiving haloperidol, 21.3% of patients receiving ketamine, 6.6% of patients receiving haloperidol plus ketamine, and 13.6% of patients receiving placebo. Again, none of the interventional study arms were statistically superior to the placebo group in preventing postoperative cognitive impairment (*p* = 0.16). Overall, the study findings do not support the use of ketamine, haloperidol, or a combination of both for preventing postoperative delirium (23).

#### Alpha-2-agonists

Dexmedetomidine, an alpha-2-agonist commonly used for sedation in the ICU, possesses anxiolytic properties with minimal analgesic effects. Studies comparing the incidence of delirium between dexmedetomidine and *γ*-aminobutyric-acid type A (GABA) modulators for sedation suggest that dexmedetomidine may be beneficial in preventing delirium (10, 24). However, there remains uncertainty as to whether these findings are incidental due to the propensity of GABA modulators to increase the risk of delirium (24). Although recent promising publications support the use of dexmedetomidine, the SCCM and PADIS guidelines do not currently recommend its use for delirium prevention (3, 4). Two articles included in this review (10, 24) specifically investigate dexmedetomidine as a prophylactic pharmacologic agent for delirium.

Su et al. (24) conducted a randomized, double-blinded, placebo-controlled trial to assess the effectiveness of dexmedetomidine in preventing delirium in elderly patients after non-cardiac surgery. The study involved 700 participants in surgical ICUs, randomized to receive either dexmedetomidine 200 μg/2 mL as a continuous intravenous infusion at a rate of 0·1 μg/kg per hour (*N* = 350) or placebo (normal saline) as a continuous intravenous infusion at a rate of 0·025 mL/kg per hour (*N* = 350) starting on the day of surgery within 1 h of ICU admission until 0800 h on the first-day post-surgery. Perioperative variables, including anesthesia methods and duration, medications administered, and surgery type, were similar in both groups. Delirium incidence and classification (hyperactive, hypoactive, or mixed) were analyzed using CAM-ICU and RASS scores. The difference in delirium incidence between the placebo and dexmedetomidine groups (22.6 and 9.1%, respectively) was statistically significant (*p* < 0.0001). Additionally, the time to onset of delirium was significantly longer in the dexmedetomidine group compared to the placebo group (6.5 and 5.8 days, respectively) (*p* < 0.0001). The occurrence of adverse effects such as bradycardia (13.1% in the placebo group and 16.9% in the dexmedetomidine group) and hypotension (26.3% in the placebo group and 32.6% in the dexmedetomidine group) were not statistically significant (*p* > 0.05). However, the incidence of adverse effects such as tachycardia (13.7% in the placebo group and 6.6% in the dexmedetomidine group), hypertension (17.7% in the placebo group and 9.7% in the dexmedetomidine group), and hypoxemia (14.3% in the placebo group and 6.9% in the dexmedetomidine group) were statistically significant (*p* < 0.002). In conclusion, the study found that the occurrence of postoperative delirium was significantly reduced in those receiving prophylactic low-dose dexmedetomidine infusion (24).

Lee et al. (10) conducted a randomized, blinded controlled trial involving 201 postoperative liver transplant patients in the surgical ICU to explore the efficacy of low-dose dexmedetomidine infusion on delirium incidence. Patients were randomized into a control group receiving 0.9% saline as a continuous infusion at a rate of 0.1 mcg/kg per hour or an intervention group receiving low-dose dexmedetomidine as a continuous infusion at the same rate. Both infusions commenced immediately after anesthesia induction and continued until 48 h postoperatively. Baseline patient characteristics did not show statistically significant differences between the groups. The incidence of delirium, assessed using the CAM-ICU score, was found not to be significantly different between the control and intervention groups (*p* = 0.44). Delirium occurred in 5.9% of patients in the control group and 9.0% of patients in the dexmedetomidine group. The duration of delirium averaged 0.8 days in the control group and 1.0 days in the dexmedetomidine group, and also showed no statistical significance (*p* = 0.51). Similarly, the average time to delirium onset (3 days in the control group and 2 days in the dexmedetomidine group) did not exhibit statistical significance (*p* = 0.37). Furthermore, perioperative bradycardia and hypotension were similar between both groups but were not statistically significant (*p* > 0.05). In contrast to the previous study, this trial concluded that intraoperative and early postoperative low-dose dexmedetomidine infusion did not decrease delirium incidence or duration (10).

#### Melatonin

Melatonin helps regulate the sleep cycle and possesses anti-inflammatory and immunomodulatory properties, which are thought to have neuroprotective effects (11). There is a hypothesized connection between poor sleep and brain inflammation in the pathogenesis of delirium (3, 4, 11).

The Pro-MEDIC (Prophylactic melatonin for delirium in intensive care) trial, a randomized, placebo-controlled, double-blind trial conducted across 12 ICUs, aimed to establish if melatonin reduces the incidence of delirium in critically ill patients. A total of 841 participants were randomized to receive either 4 mg of enteric melatonin or placebo at 2,100 h for 14 successive nights or until ICU discharge, whichever occurred first. Baseline demographics of both groups, including PRE-DELIRIC (45.3 ± 27.7 and 42.7 ± 28.8 for the melatonin and placebo groups, respectively) and CAM (40 and 35 for the melatonin and placebo groups, respectively) scores, were similar. Delirium assessment was conducted using the CAM-ICU score. The proportion of participants who developed delirium in the melatonin group and the placebo group was 35.1 and 32.7%, respectively. However, the difference in incidence between both groups was not statistically significant (*p* = 0.466). Though not statistically significant, minimal differences were observed between the groups in the percentage of patients requiring antipsychotics (21.2% in the melatonin group and 19.4% in the placebo group), sedatives (62.8% in the melatonin group and 61.1% in the placebo group), or physical restraint (14.1% in the melatonin group and 12.8% in the placebo group). No adverse events were reported in either group. Overall, the findings of the Pro-MEDIC study did not find evidence to support the use of melatonin to prevent delirium in critically ill patients (11).

#### Simvastatin

As previously discussed, neuroinflammation is hypothesized to play a role in the pathogenesis of delirium (1, 3, 4, 13). This hypothesis led to the initiation of the MoDUS (Modifying Delirium Using Simvastatin) trial, which investigated simvastatin’s anti-inflammatory properties in the prevention and management of delirium. The study involved 142 ICU patients who were randomized to receive either 80 mg of enteral simvastatin or an identical-looking placebo daily for up to 28 days, regardless of delirium status at enrollment. At baseline, 79% of participants in both groups presented with delirium based on CAM-ICU scores, while the remaining 21% were either unassessed or scored negative for delirium. Baseline characteristics and demographics were similar between groups. The incidence of delirium was comparable between the simvastatin and placebo groups (93% vs. 94%, respectively, *p* = 0·81). Likewise, there was no significant difference in the mean number of days with delirium by day 28 (6.4 days in the simvastatin group vs. 6.8 days in the placebo group, *p* = 0.80). A *post hoc* analysis of the subgroup not initially positive for delirium showed no disparity in mean delirium-free days at day 14 between the simvastatin and placebo groups. The most prevalent adverse effect observed was an increase in serum creatine kinase concentration (11% vs. 4% in the simvastatin and placebo groups, respectively), although these findings were not statistically significant (*p* = 0.208). In conclusion, the MoDUS study determined that early administration of simvastatin did not demonstrate significant efficacy in preventing or treating delirium in critically ill patients (13).

### Non-pharmacological prevention

Non-pharmacologic interventions have long been the foundation of delirium prevention, with many hospitals integrating these strategies into their protocols. The Society of Critical Care Medicine (SCCM) guidelines advocate strongly for non-pharmacologic approaches, emphasizing the reduction of risk factors through measures such as promoting regular sleep–wake cycles, minimizing invasive sensory stimulation, providing regular reorientation, and ensuring adequate pain management (3, 19). The adoption of the ABCDEF bundle has proven effective in preventing delirium and mitigating its severity in critically ill patients (3, 4).

In a single-blind, randomized controlled study published in 2018, researchers investigated the impact of bright light therapy (BLT) combined with oxygen therapy on the occurrence of delirium in 62 critically ill surgical patients. The study revealed that the combination of BLT and nasal cannula oxygen therapy significantly decreased the incidence of delirium, as determined by the CAM-ICU score. Specifically, only 2 out of 31 participants in the intervention group developed delirium, compared to 11 out of 31 participants in the control group (25).

In a 2019 randomized, controlled, parallel, and open clinical trial involving 144 participants, combined non-pharmacological interventions were shown to be effective in reducing delirium among critically ill patients. Half of the participants received standard care (*N* = 72), while the other half received a comprehensive bundle of non-pharmacological interventions, including periodic reorientation, cognitive stimulation, correction of sensory deficits (such as visual or hearing impairment), environmental management, and promotion of sleep (*N* = 72). The incidence density of delirium, as assessed by the CAM-ICU score, was significantly less in the group receiving the comprehensive bundle (1.3 × 10^–2^ person-days) compared to the control group (2.3 × 10^–2^ person-days) (*p* = 0.04) (12).

In another parallel controlled randomized clinical trial conducted by Contreras et al. (16) involving 81 critically ill patients, the efficacy of combined non-pharmacological interventions was compared to standard care. However, the non-pharmacological management in this study consisted of different methods, including spatial and temporal guidance, visual stimulus, auditive stimulus, and family support. Similar to the findings of Faustino et al. (12), the authors of this study concluded that multicomponent non-pharmacological nursing programs are superior to standard care in preventing delirium among critically ill patients. The incidence of delirium was significantly lower in the intervention group compared to the control group (5% vs. 24.4%, respectively) (*p* = 0.01) (16).

Munro et al. (17) conducted a study to investigate the role of family support in preventing delirium among critically ill patients. Thirty patients were randomized into one of three groups: receiving a family member’s recorded voice messages hourly during waking hours over 3 ICU days, receiving the same messages in the voice of a stranger, or receiving no automated reorientation messages. The study found that the mean number of days of delirium, assessed using the CAM-ICU score, was 0.3 days in the group receiving recordings in a family member’s voice, 0.6 days in the group receiving recordings in an unknown voice, and 0.9 days in the control group. These results were statistically significant (*p* = 0.0437), indicating that reorientation through automated, scripted messages, especially when recorded in family members’ voices, reduced the incidence of delirium (17).

In another randomized controlled study, the efficacy of Multicomponent Non-Pharmacological Nursing Interventions (Multi-Non-Pharma NIs) was examined in 94 patients. These interventions included orientation strategies such as playing audio recordings, reading a newspaper daily, and wearing an eye patch at night. The study aimed to determine the effectiveness of listening to an audio recording of a family member versus a non-family member. Patients were randomized into three groups: Group 1 (*N* = 30) received interventions for 3 days, including orientation messages recorded from the voice of a non-family member, reading of the daily local newspaper, and wearing an eye patch to sleep. Group 2 (*N* = 31) received the same interventions as Group 1, except orientation messages were recorded from the voice of a family member. Group 3 (*N* = 33) served as the control group and received standard care for 3 days. After the intervention period, the incidence of delirium confirmed by CAM-ICU score was 16.7% in Group 1, 6.5% in Group 2, and 27.3% in the control group. Statistical significance was found when comparing all three groups together (*p* = 0.036) and when comparing Group 2 to the control group (*p* = 0.027), but not when comparing Groups 1 and 2 to each other (*p* = 0.221). Based on these results, the authors concluded that the Multi-Non-Pharma NIs bundle involving a family voice-newspaper reading-eye patch was more effective in preventing delirium compared to standard care, consistent with the findings of Munro et al. (18).

In a randomized controlled trial involving 106 patients with severe acute pancreatitis at risk of developing pancreatic encephalopathy leading to delirium, the effects of combined comprehensive non-pharmacological interventions in the prevention of delirium were studied. The interventions included a modified Hospital Elderly Life Program (HELP), incorporating directional communication plans, cognitive therapy activity plans, and early activity plans, among others. Patients were randomized into an experimental group receiving the modified HELP regimen or a control group that received standard care alone. The study found a statistically significant difference in both the incidence (*p* = 0.033) and severity (*p* < 0.012) of delirium between the intervention and control groups. In the group receiving the modified HELP regimen, only 4% of participants developed delirium, compared to 16.98% in the control group. These findings suggest that the modified HELP program can effectively decrease both the incidence and severity of delirium in patients at risk of developing pancreatic encephalopathy (26).

In a randomized controlled trial conducted in 2019 over 12 months, a unique approach was taken to evaluate the effectiveness of multicomponent nurse-led delirium prevention strategies using a hybrid stepped-wedge cluster approach. The trial involved a large sample size of 2,566 participants across four different ICUs, with a control group (*N* = 1,184) formed using the stepped-wedge cluster design. During the study, interventions were implemented later in the study period, between the fourth and seventh months, depending on location. Participants enrolled within the first to fourth months comprised the pre-intervention group. The intervention group (*N* = 1,434) received strategies including reorientation techniques, optimization of sensory functions, environmental interventions (such as sleep management), and early therapeutic interventions (such as encouraging early mobilization). Following the introduction of the nurse-led intervention, the incidence of acute delirium was witnessed to be 10.7% (95% CI = 9.1–12.4%), compared to 14.1% (95% CI = 12.2–16.2%) during the period before the intervention, with a relative risk estimated at 0.78. However, the difference in the incidence of delirium and the number of delirium-free days between the pre-intervention and post-intervention periods was not statistically significant (*p* = 0.134 and 0.199, respectively). Overall, this stepped-wedge cluster randomized trial did not demonstrate a significant decrease in the incidence and duration of delirium among adults admitted to the ICU following the introduction of a multicomponent non-pharmacological nurse-led intervention (27).

In a study conducted by Liang et al. (28), similar to Potharajaroen et al. (25), one specific non-pharmacological delirium prevention strategy was investigated. The trial randomized 152 patients to either receive daily 30-min auditory and visual stimulation sessions or standard care for 1 week. While the difference in the incidence of delirium between the intervention group and control group was statistically insignificant (*p* = 0.71), there were notable differences in other delirium-related outcomes. The average number of delirium-free days, duration of delirium, and severity of delirium were statistically significant between both groups (*p* = 0.019, 0.004, 0.002, respectively). Specifically, the mean number of delirium-free days in the group receiving daily 30-min auditory and visual stimulation sessions was 3.66, compared to 2.84 in the control group. The mean duration of delirium in the intervention group was 1.70 days, whereas it was 4.50 days in the control group. Additionally, the average delirium severity, based on CAM-ICU scores, was 3.70 in the intervention group and 5.68 in the control group. While the study did not find statistical significance in preventing delirium through sensory stimulation, the intervention group demonstrated a significant reduction in the duration and severity of delirium, along with an increase in delirium-free days (28).

### Pharmacological treatment

#### Antipsychotics

The role of antipsychotics in the management of delirium has been long-established, but recent investigations, particularly regarding the use of haloperidol, have not provided concrete evidence to support its use (3, 4). Presently, the use of antipsychotics is not recommended in the treatment of delirium in the ICU by PADIS, except in certain specific situations where it may be warranted (4). Recent studies are exploring various approaches to the use of antipsychotics in managing delirium. Some are investigating haloperidol in conjunction with other antipsychotics (29), or other drugs such as benzodiazepines (32, 33), or investigating the newer atypical antipsychotics (30, 31) in the management of delirium.

The EuRIDICE trial aimed to assess the efficacy of haloperidol in managing delirium among adult critically ill patients in the ICU. This double-blinded trial, with 132 participants, randomly assigned individuals to either receive IV haloperidol 2.5 mg every 8 h (intervention group, *N* = 65) or placebo (control group, *N* = 67). Delirium was assessed using the CAM-ICU score. The trial was terminated prematurely due to the futility of the primary endpoint, delirium and coma-free days (DCFDs). Haloperidol administration did not result in a higher number of delirium-free days compared to the placebo (median DCFDs of 9 in both groups, *p* = 0.871). Moreover, there was no statistically significant difference in the occurrence of minimally reported adverse events between the groups. However, a notable finding was a significant reduction in the requirement for rescue benzodiazepines in the haloperidol-treated group (*p* = 0.028). Ultimately, the EuRIDICE trial did not provide evidence supporting the efficacy of haloperidol in reducing delirium among critically ill patients (14).

Hui et al. (29) conducted a 2020 double-blind, parallel-group randomized trial (*N* = 68) to explore the efficacy of antipsychotics in managing delirium among patients with advanced cancer experiencing refractory agitation. Participants admitted to a palliative care unit were randomly assigned to one of three groups: a haloperidol escalation group receiving 2 mg of IV haloperidol every 4 h, an antipsychotic rotation group receiving 25 mg of IV chlorpromazine every 4 h, or a combined antipsychotic group receiving 1 mg of haloperidol and 12.5 mg of chlorpromazine intravenously every 4 h. Treatment continued until death or discharge. Significant reductions in Richmond Agitation-Sedation Scale (RASS) scores were observed across all groups within 30 min of intervention. The average decrease in RASS within the first 30 min was −2.6, −2.4, and −2.1 in the haloperidol escalation group, the chlorpromazine group, and the combination group, respectively, with no statistically significant differences (*p* = 0.86). Similarly, the average decrease in RASS within the first 24 h was −3.6, −3.3, and −3.0 in the respective groups, also without statistical significance (*p* = 0.71). Despite the lack of statistically significant results, the study suggests the potential efficacy of high-dose haloperidol, chlorpromazine, or their combination in managing delirium and agitation, warranting further investigation (29).

In a double-blinded clinical trial investigating the efficacy of antipsychotics in treating delirium within palliative and hospice settings, 247 participants were randomized into three groups: risperidone (*N* = 82), haloperidol (*N* = 81), or placebo (*N* = 84), receiving age-adjusted titrated doses every 12 h for 3 days. Employing the NuDESC score for evaluation, both risperidone and haloperidol demonstrated higher delirium symptom scores compared to the placebo group (0.48 units and 0.24 units higher, respectively). Both the risperidone-placebo and haloperidol-placebo comparisons were statistically significant (*p* = 0.02 and 0.009, respectively). Additionally, delirium severity, as measured by the MDAS score, was significantly greater in the risperidone group compared to the placebo group, with a mean difference of 0.96 (*p* < 0.001). Participants in the risperidone and haloperidol groups experienced statistically significant adverse reactions, including extrapyramidal effects, compared to the placebo group (*p* = 0.03 and 0.01, respectively). Notably, the use of rescue midazolam per day was lower in the placebo group compared to the risperidone and haloperidol groups combined. For instance, on day 2, 16.8% of patients in the placebo group and 33.1% in the risperidone and haloperidol group combined required rescue midazolam (*p* = 0.01). This study concluded that the placebo was statistically superior to risperidone and haloperidol interventions in treating delirium within hospice and palliative care settings (30).

In a double-blinded trial comparing the efficacy of olanzapine and haloperidol in managing delirium, 98 patients admitted to medical oncology or hospice wards were randomized. Delirium severity was monitored using the DRS-R-9 scale, and outcomes such as delirium response rate (DRR) and time to response (TTR) were assessed. The DRR for the olanzapine group was 45%, whereas it was 57% for the haloperidol group, although these findings were not statistically significant (*p* = 0.23). Similarly, the average TTR was 4.5 days for olanzapine and 2.8 days for haloperidol, with no statistical significance observed (*p* = 0.18). Therapy-related adverse events were slightly more frequent in the haloperidol group (32.7%) compared to the olanzapine group (26.5%). Ultimately, delirium treated with olanzapine in hospitalized patients with advanced cancer did not demonstrate improvement in DRR or TTR compared to haloperidol (31).

#### Benzodiazepines

The use of benzodiazepines for managing agitation in delirium is a topic of contention (32). Research indicates that their administration, often employed for sedation during mechanical ventilation in the ICU, is independently correlated with a heightened risk of delirium development (3, 4).

Hui et al. (32) conducted a double-blind, parallel-group clinical trial aimed at investigating the impact of combining lorazepam with haloperidol versus haloperidol alone in managing agitation, a common symptom of delirium. This trial, conducted at an acute palliative care unit, randomized 90 patients to receive either 3 mg IV lorazepam (*n* = 47) or placebo (*n* = 43) in addition to 2 mg IV haloperidol at the onset of an agitation episode. The lorazepam plus haloperidol group demonstrated a significantly larger reduction in RASS score at 8 h (−4.1 points) compared to the placebo plus haloperidol group (−2.3 points), with statistical significance (*p* < 0.001). Additionally, the lorazepam plus haloperidol group required a lower dose of rescue antipsychotics (2 mg) compared to the placebo plus haloperidol group (4 mg) (*p* = 0.009). Caregivers perceived patients in the lorazepam plus haloperidol group to be more relaxed (84% vs. 37% for the placebo plus haloperidol group, *p* = 0.007), as did nurses (77% vs. 30% for the placebo plus haloperidol group, *p* = 0.005). The addition of lorazepam to haloperidol, in comparison to haloperidol alone, resulted in significantly greater agitation decline (32).

In a similar study conducted by Ferraz Gonçalves et al. (33) (*N* = 79), the treatment of acute agitation in inpatient palliative units was investigated, utilizing midazolam as the agent. Similar to Hui et al. (32), patients were randomized to receive either 5 mg IM midazolam (*n* = 49) or placebo (*n* = 30) in addition to 5 mg IM haloperidol at the onset of an agitation episode. The combination of midazolam and haloperidol effectively controlled 84% of agitation episodes compared to 64% using haloperidol alone (*p* = 0.002). Furthermore, the combination group demonstrated a faster resolution of agitation episodes, with an average time to control of 15 min in the combination group compared to 60 min in the control group (*p* < 0.001). Transient somnolence was reported more frequently in the combination group than in the control group. Overall, the combination of haloperidol and midazolam proved significantly more effective in treating agitation in delirium compared to haloperidol alone (33).

#### Alpha-2-agonists

The utilization of dexmedetomidine for managing delirium remains a subject of debate (3). Nevertheless, PADIS guidelines advocate for its application in certain circumstances, such as addressing agitation that impedes extubation in mechanically ventilated patients (4).

A non-randomized controlled trial investigated the efficacy of dexmedetomidine in treating hyperactive delirium unmanageable with haloperidol among 132 non-intubated ICU patients. Patients with hyperactive, agitated delirium were initially treated with haloperidol and titrated until their Richmond Agitation-Sedation Scale (RASS) score decreased to ≤0, categorizing them as responders (*N* = 86). Non-responders (*N* = 46), who did not achieve a RASS score ≤ 0 with haloperidol treatment, received additional therapy with an infusion of dexmedetomidine at 0.2 μg/kg/h, up to 0.7 μg/kg/min if necessary, to attain a RASS of 0. Following this, haloperidol was tapered and discontinued. Patients receiving dexmedetomidine spent a significantly greater amount of time under satisfactory sedation (RASS 0–2) and satisfactory Intensive Care Delirium Screening Checklist (ICDSC) scores (<4) compared to patients receiving haloperidol alone (*p* = 0.001 and 0.0005, respectively). On average, patients receiving dexmedetomidine spent 92.7% of the time under satisfactory sedation compared to 59.3% in patients receiving haloperidol alone. Similarly, patients receiving dexmedetomidine spent 52% of the time under satisfactory ICDSC scores compared to 29.5% in patients receiving haloperidol alone. Excessive sedation necessitating treatment discontinuation occurred in 10 patients receiving haloperidol, but none in patients receiving dexmedetomidine (*p* = 0.01). Although not statistically significant (*p* > 0.05), adverse effects and safety concerns were more pronounced in patients receiving haloperidol. Dexmedetomidine demonstrated significant utility in treating refractory agitation due to delirium in non-intubated patients in which haloperidol was ineffective (34).

#### Multi-component interventions targeting three neurotransmitters (acetylcholine, dopamine, and GABA)

One hypothesis concerning the pathophysiology of delirium implicates neurotransmitter imbalance (3, 4), specifically cholinergic deficiency, dopaminergic excess, and GABA overload. Based on this hypothesis, Khan, Babar A et al. devised a pharmacological management of delirium (PMD) bundle, comprising reducing exposure to 20 benzodiazepines and anticholinergics, along with prescribing low-dose haloperidol, for use in an intervention group in a clinical trial (*N* = 351). Critically ill patients in the ICU were enrolled and randomized to receive either the PMD bundle or standard care. Outcomes, including delirium-free days and delirium severity, were assessed using CAM-ICU, CAM-ICU-7, RASS, and DRS-R-98 assessments. There were no significant differences between the PMD and control groups in median delirium/coma-free days by day 30 (26 days for both, *p* = 0.991) or delirium severity at day 8. However, the intervention group exhibited statistically significant greater reductions in delirium severity compared to the group receiving standard care upon discharge (mean decrease in CAM-ICU-7 score = 3.2 vs. 2.5, respectively, *p* = 0.046). Overall, the implementation of a multi-component pharmacological bundle involving deprescribing delirium-inducing medications alongside prescribing low-dose haloperidol did not reduce delirium among critically ill patients but may yield positive outcomes later in the course of hospital care (15).

#### Opioids, anticonvulsants, and analgesics

It is well established that timely identification and management of pain significantly influence the development and management of delirium (3, 19). However, many commonly used pharmacologic agents for analgesia can also induce or perpetuate delirium symptoms, including hallucinations (19). A randomized controlled trial involving 352 nursing home residents discovered that implementing a stepwise protocol for pain treatment (SPTP), comprising paracetamol, morphine, buprenorphine, or pregabalin, not only alleviated pain but also reduced delirium symptoms (assessed using the NPI-NH). Importantly, opioid analgesics did not lead to an increase in psychotic symptoms. The intervention group demonstrated statistically significant superiority over the control group in reducing average scores for delusions (−4 vs. −2.4, respectively, *p* = 0.031) and agitation (−2.6 vs. −0.5, respectively, *p* = 0.004). This study highlighted that the use of paracetamol, morphine, buprenorphine, or pregabalin did not exacerbate delirium symptoms and, conversely, alleviated the prevalence of delirium by addressing pain management (7).

#### Simvastatin

See “Simvastatin” under the Pharmacological Prevention section.

## Discussion

This comprehensive review examined the efficacy of pharmacological and non-pharmacological interventions in the prevention of delirium and pharmacological interventions in the treatment of delirium. Twenty-six articles were included in the review (*N* = 8,831) that investigated pharmacological prophylaxis (*N* = 9), non-pharmacological prophylaxis (*N* = 8), and pharmacological management of delirium (*N* = 10).

Preventing delirium in critically ill hospitalized patients remains pivotal. Guidelines advocate for limited pharmacologic use, favoring multicomponent non-pharmacologic strategies like the ABCDEF bundle. Emerging research not only supports the use of non-pharmacologic methods but also highlights the efficacy of family member support and bright light therapy, specifically. The role of medications in prevention remains uncertain. While traditional agents like haloperidol demonstrate poor prophylactic efficacy, newer atypical antipsychotics such as aripiprazole and quetiapine show promise. However, agents like melatonin, ketamine, simvastatin, and dexmedetomidine show mixed or ineffective results.

Historically, haloperidol was the primary treatment for delirium, but recent studies question its continued use. Chlorpromazine emerges as a potential alternative, either alone or in combination with haloperidol. Olanzapine and risperidone show limited efficacy. Concerns surround the use of benzodiazepines due to sedative effects exacerbating delirium, though studies suggest favorable outcomes when combined with haloperidol, mitigating delirium severity and duration without significant somnolence. Similarly, opioid use’s role is contentious due to potential delirium exacerbation, yet recent studies suggest that a stepwise approach to pain management that includes opioids can improve delirium outcomes without significant side effects. Effective pain management, often involving opioids, is integral to delirium management.

### Study strengths, limitations and future studies

This review possesses several strengths. First, it offers a comprehensive overview of a substantial sample size of evidence covering various non-pharmacological and pharmacological interventions, including unconventional strategies such as bright light therapy or melatonin, not only for delirium prevention but also for management. Additionally, it meticulously evaluates the strength of evidence presented in each study and assesses potential biases. Furthermore, the review includes an analysis of side effects associated with pharmacologic interventions, enhancing its clinical relevance. Finally, it exclusively incorporates randomized controlled trials (RCTs) and non-RCTs with active comparators, placebo, or no treatment, thus mitigating the inherent bias risk often associated with observational studies.

However, this review also has several limitations. Due to the inclusion of both RCTs and non-RCTs with varying methodologies, medications, and outcomes, a quantitative analysis of the effects of these interventions on delirium outcomes was not conducted. Additionally, many of the interventional studies had notable limitations of their own, including lack of blinding, inadequate or absent randomization methods, and small sample sizes. Finally, some of the included intervention trials were not prospectively registered, or the registration process was not adequately described in the original research articles.

To advance delirium management, meticulously designed multicenter, double-blinded, placebo-controlled, adequately powered trials are imperative. Such trials should evaluate the impact of pharmacologic agents on delirium prevention and treatment across various patient populations and treatment settings. This approach will equip clinicians with the necessary evidence to make informed decisions regarding the prevention and management of delirium symptoms in high-risk populations.

## Conclusion

In conclusion, the primary focus of delirium management lies in prevention. Non-pharmacologic strategies, especially family support, are the most effective at preventing delirium in critically ill hospitalized patients. Though the evidence is somewhat mixed, there is evidence that specific medications, including atypical antipsychotics and dexmedetomidine, can decrease the incidence of delirium among hospitalized patients. Pharmacologic agents as remedies for delirium have limited significant evidence, but promising results have been observed using opioids for pain management and the combination of benzodiazepines and haloperidol, particularly in cases of hyperactive delirium with agitation. Therefore, we would recommend the use of these medications, when necessary, in addition to non-pharmacologic interventions and addressing the underlying conditions. The saying, “the best treatment is prevention” rings particularly true in the context of delirium management.

## Author contributions

LC: Conceptualization, Data curation, Formal analysis, Funding acquisition, Investigation, Methodology, Project administration, Resources, Software, Supervision, Validation, Visualization, Writing – original draft, Writing – review & editing. GC: Supervision, Writing – review & editing.
